# Annotations

**Published:** 1911-09-23

**Authors:** 


### Annotations.

### The Cost of a Strike.

There is a gruesome little French play called "Sabotage." It tells of a workman on strike who has gone out, leaving his wife to look after their sick child. The little flat where the scene of the play is laid has many modern comforts—among them electric light. The doctor comes to see the child and finds that an immediate operation is necessary. He is preparing for it when the light goes out. Before the mother can borrow a lamp or candle —so unnecessary in the usual run of her daily life— the child is dead, for the operation cannot be done in the dark. And upon her mourning enters her husband, proud and elated; he and his companions had persuaded the electric-light workers to come out on strike; they had "saboté" the light. Our recent industrial troubles have forced on us what a general strike means. There have been dislocation of business, endless annoyance, and irritation. But this has been the least of the effects. Of the obvious ill results the newspapers have been full; but of more importance is a statement by the Home Secretary, that the death-rate of children in Liverpool has been nearly doubled. What children are those? Not the children of the rich, or those who can, even at an enhanced price, obtain the necessaries of life, but the children of the poor, the children of the strikers and their comrades. The class who strike are also those who suffer most. At the best the loss of a week's wages bears hardly on the poor. If a man who earns £1 a week demands an extra shilling, and gets it after only one week's fight, it will take him twenty weeks at the increased wage to make up for the one week lost; his advantage will not begin to appear until that time is over. The advantage may be worth fighting for—one dare no more say that a strike is always wrong than that a war is always wrong. But war, military or industrial, means waste, means loss which it will take long to make up; there remains always to be considered the loss for which there is no compensation—the death of the children of the poor, the permanently impaired vitality of those who, having gone through the hardships of a strike, manage to survive. It may be right—it may be heroic to grudge "nor limb, nor life, nor child, nor wife" in the cause a man has at heart, but it is well to realise the price before incurring it. That is where sabotage fails.

### The Sunday Pump.

The debate in a rural council as to whether a pump should or should not be closed on Sunday brought to light different views as to the desirability of keeping water. One councillor contended that water kept over Sunday was not so pure and wholesome as water drawn fresh from the pump. Another councillor ridiculed the idea. The gentleman who considered fresh water to be preferable is no doubt the more likely to be correct. Our ordinary tap water contains a considerable number of micro-organisms, and the water from the greater number of village pumps probably does not compare too favourably with the supply of water companies. Should the water from the pump happen to contain any traces of organic matter the micro-organisms within it would multiply rapidly. In such a case there can be no doubt that water kept for twenty-four hours is more likely to present an element of danger than water freshly drawn.

### The Cholera in Italy.

A good deal of public alarm has been aroused, more particularly among those who travel abroad, by the present outbreak of cholera in Italy. So far there seems no ground for any anxiety as the epidemic has been detected, and will probably be held in check without difficulty. It must be remembered that in Turkey and in Russia cholera has been practically endemic for some time. It is therefore not astonishing that small outbreaks should occur outside the limits of those countries; and the fact that whenever they do they are always promptly suppressed is a fairly good proof that modern sanitation, as evolved in the more civilised States of Europe, is equal to the task of confining the cholera to those less forward countries where dirt diseases still flourish unhindered. Those who are thinking of travelling in Italy may be advised that there is practically no risk of catching cholera, especially if they follow the usual custom of visitors to that country by drinking mineral waters or wine throughout their stay. With such centres of cholera diffusion as are provided annually by St. Petersburg and other towns at this time of year, it is to be wondered at that more sporadic outbreaks do not occur in neighbouring countries.

### Death in Bed.

The majority of cases of death in bed occur in elderly or late middle-aged people. They are undoubtedly instances of cardiac failure. Why death should thus be common at times in which little strain is thrown upon the heart may not be very obvious, but that the heart is at fault cannot be doubted. We hear, however, also of the possibility of suffocation taking place during a nocturnal attack of epilepsy. During or just after the fit the person attacked is said to turn the face into the pillow, in which position suffocation takes place. The death recently recorded of a lady novelist at the comparatively early age of thirty-seven was possibly of this nature. It was thought that while leaning out of bed she was seized with a fainting attack, and that, judging from the position of her head, she was subsequently smothered. Fainting attacks, needless to say, are as often as not of the nature of epilepsy. The medical man who attended said that the lady had "frequently had fainting attacks during which she would lose consciousness for a short period." Such fainting attacks were probably epileptic in nature. It is doubtful whether, in an ordinary faint, reflex action and power of movement would be abolished for sufficient length of time to allow of suffocation taking place.

